# The complete mitogenome of *Saccharina* cultivar ‘Haiyi No.1’ (*Saccharina japonica* × *latissima*) and the phylogenetic analysis

**DOI:** 10.1080/23802359.2019.1704644

**Published:** 2020-01-09

**Authors:** Pengjun Liu, Jing Zhang

**Affiliations:** Qilu University of Technology (Shandong Academy of Sciences), Jinan, People’s Republic of China

**Keywords:** Cultivar ‘Haiyi No.1’, mitogenome, phylogenetic relationship

## Abstract

Here, we reported the complete mitogenome of *Saccharina* cultivar ‘Haiyi No.1’ (*Saccharina japonica* × *latissima*). This mitogenome had a circular mapping organization with the length of 37,657 bp and contained 66 genes including 35 protein-encoding genes, 3 rRNAs, 25 tRNAs, and 3 open reading frames (ORFs). The AT content accounted for 64.7% of the entire mitogenome. The gene content and gene order were consistent with those reported *Saccharina* species and cultivars. Phylogenetic analysis showed the phylogenetic relationship among Chinese *Saccharina* cultivars and demonstrated that ‘Haiyi No.1’ had a closer relationship with *Pingbancai* which strongly supported its genetic source.

*Saccharina* (Laminariales, Phaeophyceae) species are the economically important brown seaweeds (Kain 1979). There are more than 20 *Saccharina* cultivars been bred in China which has contributed significantly to the mariculture. ‘Haiyi No.1’ bred from the ‘Pingbancai’ which is an intraspecific hybrid of ‘Fujian’ (*Saccharina japonica*) and ‘901’ (*Saccharina japonica*×*latissima*). At present, more than 10 mitogenomes of Chinese *Saccharina* cultivars have been completed. However, genomic information of ‘Haiyi No.1’ is limited. Here, we characterized the complete mitogenome of ‘Haiyi No.1’ and constructed phylogenetic analysis to provide new molecular data for the molecular study.

‘Haiyi No.1’ individuals were collected from Rongcheng, Shandong Province, China (37°15′39′′N, 122°33′56′′E), and then specimen (sample accession number: 2016070089) was deposited in the Culture Collection of Seaweed at the Ocean University of China. Subsequently, the homologous PCR amplification and primer walking strategy were used (Zhang et al. 2019).

The ‘Haiyi No.1’ mitogenome comprised a circular molecule of 37,657 bp (GenBank accession number MG712778, https://www.ncbi.nlm.nih.gov/nuccore/MG712778.1/), with a nucleotide composition of 28.4% A (10,695), 14.7%C (5536), 20.6% G (7757), and 36.3% T (13,669), and had an overall AT content of 64.7%. This mitogenome encoded 3 rRNA genes (*rnl*, *rns*, and *rrn*5), 25 tRNA genes, 35 protein-encoding genes, and 3 ORFs (*orf*41, *orf*130, and *orf*377). The protein-encoding regions were 28,667 bp in length accounting for 76.1% of the whole mitogenome. Among these 66 genes, only *rpl*2, *rpl*16, *rps*3, *rps*19, *tat*C, and *orf*130 were encoded on L-strand. The universal genetic codes were used and all start codons were ATG. Of the 35 protein-encoding genes and 3 ORFs, 26 (68.4%) terminated with TAA codon, higher than that for TAG (9, 23.7%) and TGA (3, 0.08%). Moreover, all tRNA genes were provided with standard clover-leaf secondary structures. Gene content and gene order showed a high level of conservation with those reported *Saccharina* mitogenomes (Yotsukura et al. 2010; Zhang et al. 2013; Zhang et al. 2017; Zhang et al. 2018; Zhang et al. 2019).

A comparison of the complete ‘Haiyi No.1’ mitogenome with that of ‘Pingbancai’ (Accession number: KX073817) showed there were only three mutation sites which indicated high conservatism at the intergenic region.

Bayesian analysis based on the whole mitogenomes of the 18 *Saccharina* and *Laminaria* algae was conducted to reconstruct the phylogeny (Figure 1). *Ectocarpus siliculosus* served as the out-group. Phylogenetic analyses showed that all algae were divided into two branches: *Saccharina* and *Laminaria*. All Chinese cultivars including ‘Haiyi No.1’ belonged to the *Saccharina* lineage. ‘Haiyi No.1’ first clustered with ‘Pingbancai’, ‘Zaohoucheneg’ and ‘Ailunwan’ supporting its genetic origin. The phylogenetic tree in this work also provided more information for the current understanding of genetic relationships among Chinese *Saccharina* cultivars.

**Figure 1.**
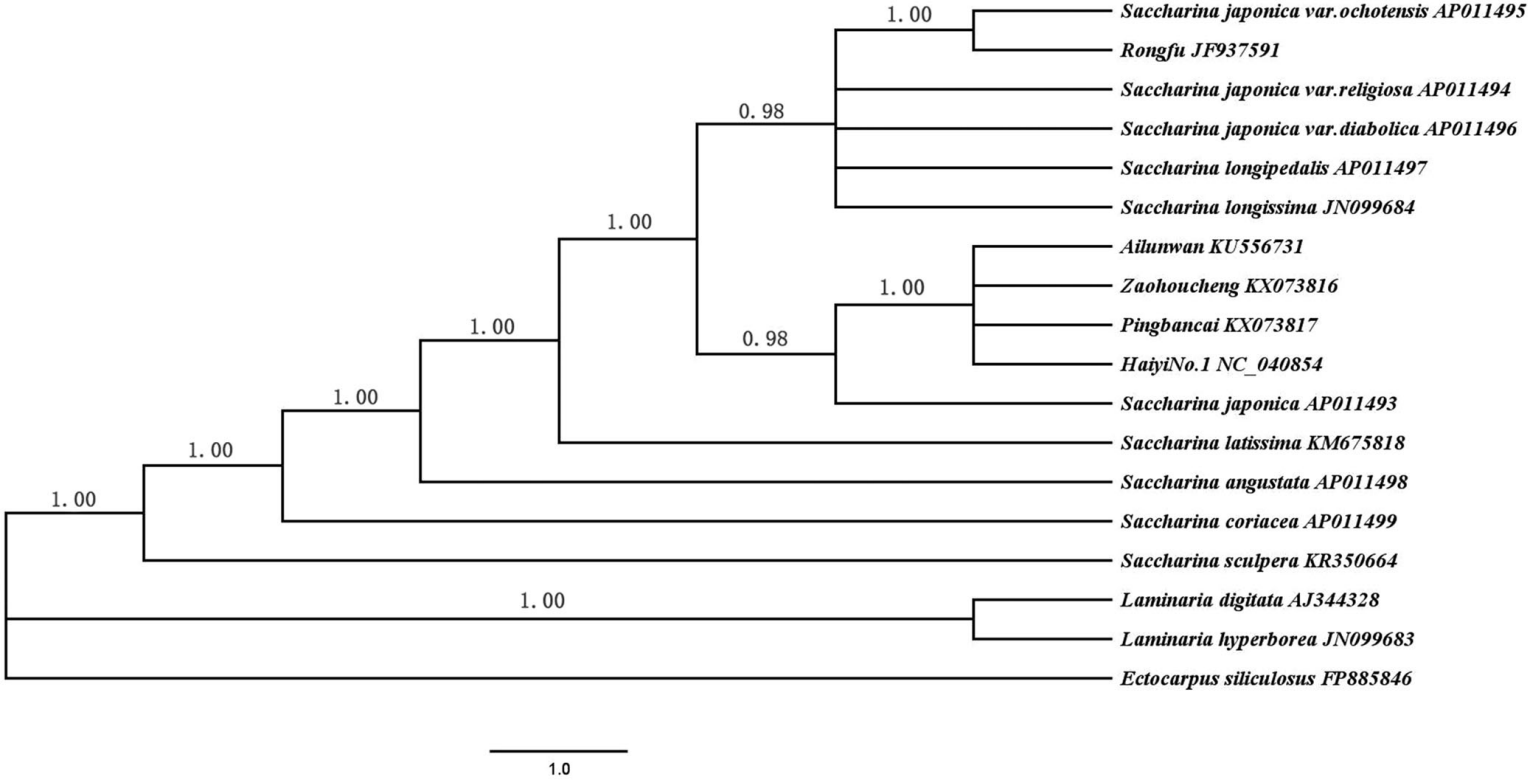
Phylogenetic tree constructed based on combined 35 mtDNA protein-encoding genes using Bayesian analysis.
